# The health experience of children, adolescents and their families during the COVID-19 pandemic: an exploratory qualitative study in pediatric homecare

**DOI:** 10.3389/fped.2025.1492433

**Published:** 2025-06-20

**Authors:** Clélia Zahnd, Sandra Zoni, Marie-Aline Gonthuey, Julie Bucher Andary, Véronique de Goumoëns

**Affiliations:** ^1^ La Source School of Nursing, HES-SO University of Applied Sciences and Arts Western Switzerland, Lausanne, Switzerland; ^2^ Home-Care Services, Association Vaudoise d’Aide et de Soins À Domicile, Lausanne, Switzerland

**Keywords:** COVID-19, SARS-CoV-2, pediatric, homecare, family-centered care, pandemic, health experience

## Abstract

**Introduction:**

The COVID-19 pandemic has had a significant impact on society. Families with an ill child were more vulnerable to that context. To the best of our knowledge, no study has explored the health experience of the entire family during the COVID-19 pandemic by giving them a voice. Thus, our study aimed to explore the health experiences of children and adolescents aged 11 years and older, as well as their families, who received pediatric home care in the canton of Vaud, Switzerland, during this pandemic for an initial health problem or as part of ongoing care.

**Methods:**

A qualitative approach was employed, including 27 semi-structured interviews (but for quality reasons only 25 were coded) with children and adolescents aged ≥ 11 years and their family members who received pediatric home nursing care in the canton of Vaud. The interview guide was based on the Calgary Family Assessment and Intervention Model. Data were collected from February to April 2023 and analyzed using an inductive and deductive approach based on the theoretical framework. The total duration of the interviews is 958 minutes, and they lasted between 15 and 80 minutes.

**Results:**

The findings highlight that families with an ill child face numerous challenges at individual, familial, and community levels. They were perceived heterogeneously between the participants. For instance, government measures were sometimes perceived as precious resources and sometimes not. While some challenges are exacerbated by the pandemic, others are unrelated. However, it is important to emphasize that these families also possess a variety of resources that stem from the same systems levels. Practical needs as prioritized access to food delivery were highlight by parents, specially mothers who seemed to support the majority of the burden.

**Conclusions and implications:**

Despite their remarkable resilience, families experienced difficulties during the COVID-19 pandemic. This underscores the need to learn from this experience to prepare for the future better. some measures must also be quickly implemented to counter the long-term deleterious effects of the pandemic, especially regarding the health of children and adolescents particularly in terms of psychosocial support for families. A better focus should be made on siblings to take care of them as they are to often the great forgotten ones.

## Background

In Switzerland, an estimated 10% of children and adolescents live with chronic diseases, such as diabetes and asthma, or are at risk of developing these diseases (1). This number increases when considering children with acute illnesses. The presence of an ill child in a family has a significant impact on its functioning (2), as well as on the health of the siblings in terms of quality of life (3) and the risk of experiencing depression (4). As the consequences involve the entire family, their involvement is crucial in both clinical practice and research (5).

In March 2020, the World Health Organization (WHO) officially designated coronavirus disease caused by the SARS-CoV-2 virus (COVID-19) as a pandemic (6). This global health crisis has had a profound and far-reaching impact on individuals worldwide, giving rise to significant societal changes in the short, medium, and long term. To date, over 670 million cases and nearly 6.8 million deaths have been reported globally (7). In Switzerland, the pandemic has affected more than 4.4 million individuals, resulting in approximately 14,200 deaths (8).

The prompt implementation of health crisis management (see Table 1) measures has had substantial impacts on the psychological, social, and health aspects of the population, in addition to the deaths caused by the virus itself and the potential long-term neurological and metabolic consequences (9, 10). Isolation, social distancing, protective measures, and economic closures presented daily challenges to individuals, families, communities, and society.

**Table 1:** Measures during the two first waves of COVID-19 in Switzerland.

Measures during the two first waves of COVID-19 in Switzerland	
First Wave (11)	
Mid-March 2020	Cases initially limited to specific groups and health care professionals.
	Extension to the entire population
	Emphasized the importance of personal hygiene, testing and contact tracing.
	Public was encouraged to stay at home, (“semi confinement”). Not mandatory.
	Schools, stores (excluding those selling essential items), and recreational facilities were all closed.
April 2020	Reopening of health care facilities and some stores.
May 2020	Reopening of schools and all stores.
June 2020	Reopening of recreational facilities in June.
Second Wave (11, 12)	
July 2020	Masks have to be worn in public transport.
October 2020	Masks have to be worn in closed public space.
Mid-Decembre 2020	Restaurants, bars, cultural and sports venues had to close again.
	Beginning of the vaccination for vulnerable people.
	Next, teleworking became obligatory, and establishments that did not sell necessities closed their doors.
April 2021	Cultural and sports venues and restaurants reopened, but restrictions on the number of people who could access them were implemented.
	Afterward, COVID-19 certificates and vaccination have played a major role (11).

The COVID-19 pandemic has had a significant impact on families with children who have preexisting health issues, such as developmental disorders related to autism spectrum disorder, congenital lesions, or attention deficits. These families reported a significant increase in the caregiving burden (13); a high prevalence of depression (62%), anxiety (20%), and stress symptoms (36%); and an impact on the continuity of care (14). Therefore, ensuring health care for sick children despite sanitary restrictions is of paramount importance for the entire family's well-being. Nevertheless, parents' indispensable role in the recovery process of a child is indisputable. In 2019, a meta-analysis highlighted the benefits of family nursing interventions on parents' depression, anxiety, satisfaction, and stress (15). However, the pandemic has transformed the relationship between the population and health care structures. A study showed that families whose children required occasional care during the pandemic were very hesitant to go to the hospital; of the 35% of parents who reported that their child needed care, only 22% went to the hospital (16). Another study reported that 85% of parents of children with cancer were afraid of an infection due to immunosuppression, and 70% perceived the hospital as a dangerous environment, worried about suboptimal oncological care and reported the psychosocial and economic impacts of isolation on their families (17, 18). In that context, home care was a really important resource. This is why this study focused on the experiences of the families whom a child has a chronic disease and who received home care during the COVID-19 pandemic.

Home care is highly appreciated by both children and families. Two literature reviews exploring the experiences of children and their families during home care highlighted a growing attraction to this type of service, providing them with more freedom, especially for social and professional activities, better family management, and increased involvement in exchanges and decision-making regarding disease management (19, 20). Children and families emphasize the importance of considering their needs and establishing a strong alliance with health care professionals to ensure effective and satisfying health care (19, 21).

Our study is rooted in the theoretical framework of family systems nursing (FSN) (22–24). FSN views the individual and their family as a unit of care, recognizing that when one family member experiences a health issue, the entire family must be considered. Health experience is defined as the subjective nature and unique way in which an individual or family perceives health-related events, encompassing experiences encountered in contexts such as growth, development, and illness (25). The family is defined as a group of individuals bound by deep attachment and a sense of belonging, where each person identifies as a family member (23, 26). Emphasis is placed on the dynamics of interaction and reciprocity, with an approach of “both/and” rather than a binary perspective of “either/or”. Family nursing interventions have shown benefits for patients with serious illnesses, such as cancer. According to a systematic review by (27), family interventions affect the self-efficacy of caregivers and, more broadly, the overall quality of life of the entire family. Although family-centered care has been implemented in pediatric care for a long time, it is neither systematic nor universal, and uniformity in methodological terms is lacking (28).

Currently, limited research is available regarding the disruptions caused by the pandemic on the health of family members, both in Switzerland and internationally. A qualitative study explored the experiences of mothers only (29), a survey described the experiences of parents (30), and a qualitative study explored the experiences of clinicians (31). Studies of parents and mothers have shown heterogeneous results. The COVID-19 pandemic is a source of stress and fear for mothers of people with intellectual disabilities (29) but also has an impact on health and well-being of children with chronic diseases (31). On the other hand, Cadwgan et al. (2022) reported a more effective functioning of the hospital services during the pandemic than before the pandemic, in the way patients are cared for, depending on their situation. Other studies have explored the use of telemedicine, for example, to treat asthma (32) or children with developmental disabilities (33). To the best of our knowledge, to date, no study has explored the health experience of the entire family during the COVID-19 pandemic by giving a voice to the children, their siblings, and their parents. Thus, our study aimed to explore the health experiences of children and adolescents aged 11 years and older, as well as their families, who received pediatric home care in the canton of Vaud, Switzerland, during the COVID-19 pandemic for an initial health problem or as part of ongoing care.

## Methods

### Research design

This study used a qualitative exploratory design involving semi structured individual interviews in an interpretative paradigm. The authors adhered to the Standards for Reporting Qualitative Research (SRQR) (34) (see Supplementary Appendix 1). The study was approved by the human research ethics committee (CER-VD 2019-00825).

### Study setting and participants

The study was carried out in a pediatric homecare service that cares for children aged 0-18 suffering from a variety of health problems. Over 400 children and their families are being cared for in the canton of Vaud throughout the yearly. These children suffer from oncological, hematological, endocrinological, neurological, rheumatological and psychiatric pathologies. Eligible participants were identified in close collaboration with the partnering field team through an internal database based on the inclusion and exclusion criteria mentioned below. They were subsequently contacted by the research team (CZ, SZ and VdG) to provide them with the requisite information to make an informed decision about their participation. At least 24 h before data collection, the participants were provided with an information sheet and a consent form. The participants received a gift card to thank them for their contributions.

### Inclusion/exclusion criteria

The target population included children or adolescents aged ≥11 years who received home care for more than 30 days during the pandemic (March 2020–March 2022) for an initial health problem or as part of ongoing care, and their family members. A convenience sampling has been used for this research as the target population is difficult to reach. The participants had to understand and speak French and to be physically able to speak. They also had to sign the consent form and agree to be recorded. For children under 14 years old, the consent form had to be signed by their legal representatives. Otherwise, or in the event of the child's death, they did not participate in this study.

### Data collection

The semi structured individual interviews were conducted by the research team in person, either at the participants' homes or at the requesting institution, between February 2023 and April 2023. They were recorded on a digital recorder with the agreement of the participants. Children under 14 years of age could participate alone or with a family member, while parents could decide to allow their children to participate alone or accompany them during the interview. Children over 14 were interviewed alone, after giving their consent. The individual interviews enabled each family member to express their own experiences individually and provided greater flexibility in scheduling interviews based on the activities of each family member. Although a focus group was offered, it was not successful among the participants who preferred individual interviews. We used an interview guide that was developed by the research team and grounded in theoretical foundations (see Table 2). The sentence structures were simplified for the younger participants, aged between 11 and 14. Each interview lasted between 15 and 80 min.

**Table 2:** Guide for the semi-structured interviews.

Type of question	Specific question
Main exploratory question	The pandemic has had a major impact on society as a whole. Can you tell us about your personal experience in caring for yourself or your child/relative (brother, sister, grandchild, etc.)?
Follow-up question on temporality	How did you experience with the different waves and the measures put in place?
Follow-up question on barriers to family health	What have been the most difficult moments related to your/your child's/your loved one's health during this pandemic?
Follow-up question on the future	Looking ahead, is there anything you're particularly afraid of?
Follow-up question on the factors that facilitate family health	What means have you put in place to cope with the pandemic and continue to take care of your/your child's/your loved one's health?
Follow-up question on changes in family health	If you think about your health or the health of your child and family, what major change do you think has taken place in your family during the pandemic?
Follow-up question on the relationship with nursing teams	When do you think you most needed home nursing teams? Or when would you have needed home nursing teams most?
Follow-up question on emotions	When you say that [you've been scared/angry/stressed] how does that manifest itself in your body or in your head? Or you tell me you've been [anxious, stressed] can you tell me a little more about that?
Follow-up question on exploring family functioning	How did “other” family members experience this situation? When you say that [your child/family member] has experienced this/said this, how do you think others feel about it?
Follow-up question on external resources	Apart from the family members mentioned, were there any other people who particularly supported or helped you in this situation?

Prior to the interview, participants were provided with a demographic questionnaire. The questionnaire included details pertaining to gender, age, native language, educational attainment, perceived socioeconomic status, self-reported health condition, and, for family members, their relationship with the child or adolescent receiving care. Additionally, specific medical information was obtained, including information on the health issues that necessitated home care for children or adolescents, as well as any existing medical diagnoses. Conforming to the theoretical framework, the family structure was delineated through the construction of a family genogram.

Data collection continued until no new information emerged in the interviews (data saturation), which was determined through a coding matrix-based grid, following the recommendations of the literature (40).

### Data analysis

The data were analyzed by at least two members of the research team using MaxQDA 18.0 software (35). A third member of the research team compared the analyzes and settled when the coding differed. The thematic content analysis method (36) was used for this research, with a coding matrix developed by one of the researchers (VdG) in a previous study (37) and adapted with an inductive approach to fit the present data. The original matrix was grounded on the Calgary model (23).

The sociodemographic data, such as the means, standard deviations, minima and maxima, were included in the descriptive analysis.

### Criteria for scientific rigor

The criteria for scientific rigor and the reliability of the research data (trustworthiness) were met using the principles outlined by Lincoln and Guba (1985) (38, 39). The credibility of the data was ensured through regular communication between the research and field teams. However, we were unable to present the results to the participants; although we invited them to a meeting at the requesting institution, only one responded and declined. Additionally, the analyses were initially performed independently by at least two members of the research team and then aggregated to minimize potential biases in understanding and interpretation. The research team included varied expertise such as a pediatric nurse, a psychologist and a researcher with an extend experience in family systems nursing research, practice and education.

The research process was conducted with the utmost transparency in both data collection and analysis to ensure the confirmability of the data. The participants were clearly explained the purpose of the project during the first contact by phone and in person before the interviews. The research team prioritized data quality over quantity, and therefore only trained individuals familiar with this data collection method were included in the research team to ensure high-quality data during the interviews. Data analysis was conducted simultaneously with the data collection process, enabling the research team to verify the achievement of data saturation. The principle of transferability was ensured through a detailed description of the context and participants. Reflectivity was ensured by maintaining a study journal.

### Research findings

A total of 27 interviews were conducted between February 13 and April 27, 2023, in the canton of Vaud, with a total duration of 958 min. The interviews lasted between 15 and 80 min. These interviews resulted in 1,798 coded segments.

### Sample description

A total of 27 participants were interviewed for this study. The participants were divided into three groups: Child (referring to children or adolescents who received care; *n* = 10), Sibling (*n* = 2), and Parent (*n* = 15). The mean age of the Child group was 16.3 years (SD = 2.26; min = 12, max = 20), the mean age of the Sibling group was 19.5 years (SD = 0.71; min = 19, max = 20), and the mean age of the Parent group was 47.2 years (SD = 5.94; min = 34, max = 56). Table 3 provides detailed sociodemographic results.

**Table 3:** Detailed socio-demographic results depending on categories (child, sibling and parent).

Participants	Child	Sibling	Parent
Total number of participants *n* (%)^a^	10 (37,04)	2 (7,41)	15 (5,55)
Age *Mean (SD)*	16,3 (2,26)	19,5 (0,71)	47,2 (5,94)
Genre *n (%)*			
Masculine	4 (40)	1 (50)	5 (33,3)
Feminine	6 (60)	1 (50)	10 (66,6)
Formation *n* (%)			
Federal Certificate of Professionnal Training	0 (0)	0 (0)	1 (6,66)
Bachelor/Master/Doctorat	0 (0)	0 (0)	8 (53,33)
Federal Certificate of Competence	0 (0)	0 (0)	2 (13,33)
Federal diploma	0 (0)	0 (0)	2 (13,33)
Baccalaureate School	3 (30)	1 (50)	0 (0)
Specialized Baccalaureate	0 (0)	1 (50)	0 (0)
Compulsory education	6 (60)	0 (0)	2 (13,33)
Other	1 (10)	0 (0)	0 (0)
Mother tongue*^b^ *n*			
French	7	1	12
English	4	2	2
Portuguese	1	0	2
Spaniard	1	0	2
Bengali	0	0	1
Serbo-Croatian	1	0	1
Perceived Economic Status *n* (%)			
Comfortable	7 (70)	2 (100)	8 (53,33)
Sufficient	3 (30)	0	1 (6,66)
Insufficient	0	0	6 (40)
Self-reported health status *n* (%)			
Good	8 (80)	2 (100)	14 (93,33)
Moderate	2 (20)	0	1 (6,66)
Weak	0	0	0
Relationship with the child *n* (%)			
Mother	NAP	0	9 (60)
Father	NAP	0	5 (33,33)
Stepmother	NAP	0	1 (6,66)
Brother	NAP	1 (50)	0
Sister	NAP	1 (50)	0
Lives with the child *n* (%)			
Full-time	NAP	2 (100)	12 (80)
Part-time	NAP	0	3 (20)
No	NAP	0	0
Type of child health problem* *n*^c^			
Related to a chronic disease	7	2	12
Related to mental health	1	0	3
Developmental or disability-related	1	0	3
In connection with an accident or acute illness	2	0	4

^a^ The percentage is calculated on the total number of participants. For the rest of the year, the percentage is calculated according to the number of participants per category.

^b^ * = Multiple responses possible per participant.

^c^ For the children, the diagnoses announced were reclassified in the categories by the researchers.

### Analysis of interviews

**Figure 1:**
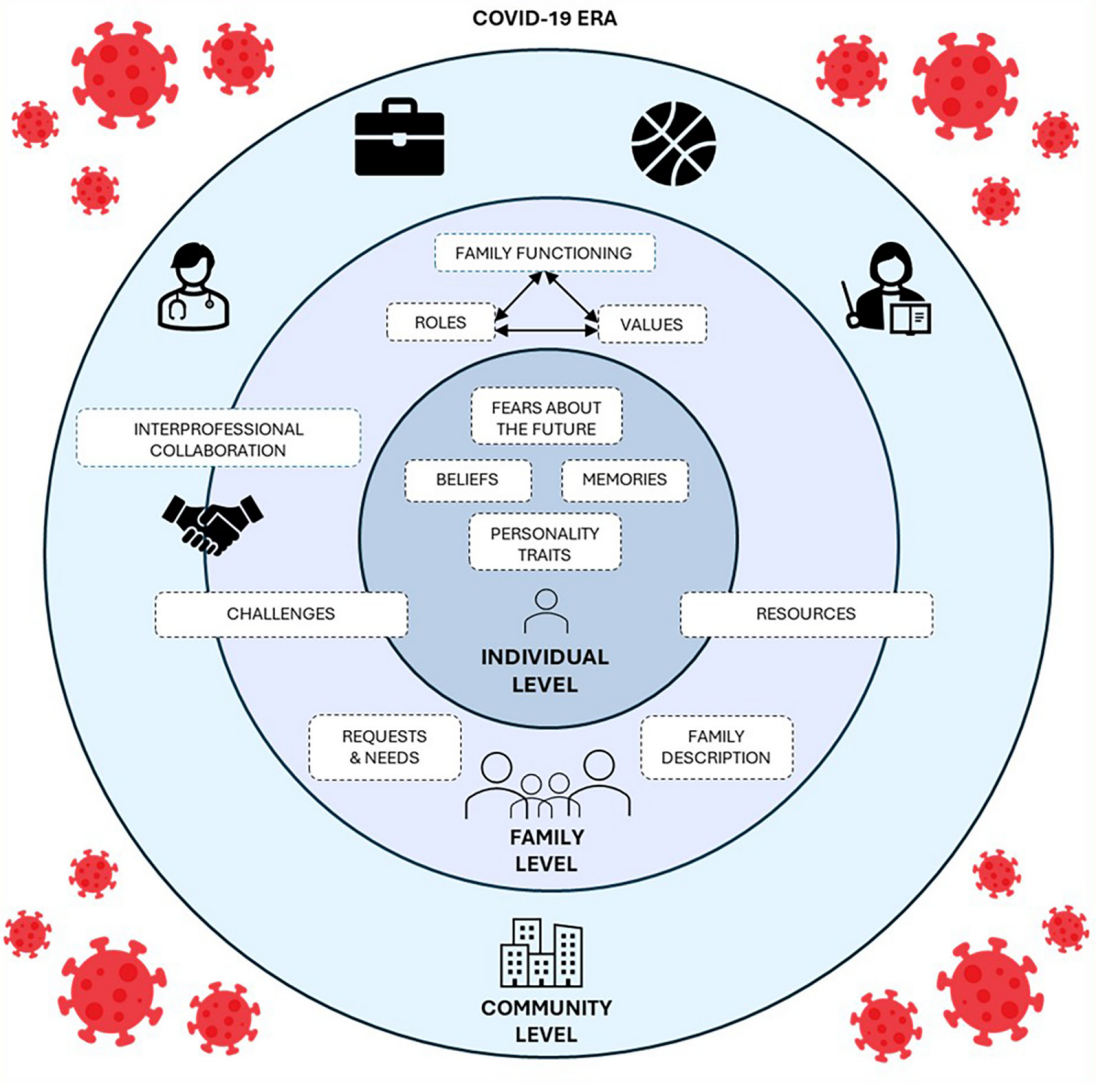
Summary of results.

For quality reasons (technical difficulties and verbal competencies), only 25 interviews were coded for the analysis. The analysis of the results revealed seven predominant categories influencing the health experiences of individuals and families during the pandemic (family system, nursing team system, nursing interventions, environment, collaboration, fears about the future, and experience/memories of the COVID-19 pandemic), along with 24 subcategories (see Supplementary Appendix 2). Figure 1 shows a synthesis of the findings.

#### The health experience during the COVID-19 pandemic: challenges and resources for families

The interviews revealed themes that influenced health experiences during the COVID-19 pandemic at the individual, familial, and community levels. Participants' health experiences encompassed both challenges and mobilized resources at every level. In general, this study highlights the ambivalence of the experiences between families and between individuals inside the same family. COVID-19 was a specific period with its own challenges and resources but was also in some cases a really secondary concern.

### Individual level

This study delved into the beliefs and perceptions of participants regarding health crises. Some participants highlighted a form of disbelief in the global health crisis: two individuals expressed doubts about the decisions made. Nevertheless, most participants appreciated the health directives implemented by the government, which facilitated their decisions. Many participants shared their beliefs about the role of the media and perceived a negative impact on the pandemic experience. They emphasized the media's focus on the death toll and daily dissemination of bad news, eliciting negative emotions. Furthermore, several participants chose to isolate themselves to protect either themselves or their loved ones, using terms such as “isolation,” “self-sufficiency,” “living under a bell jar,” or “living in a bubble.”

Moreover, both parents and children found it challenging to return to school after the lockdown. The participants expressed negative emotions, such as sadness, guilt, anger, and even depressive states. The parents found understanding what their children were going through difficult and highlighted the suffering of their teenagers. This situation occasionally led to intergenerational conflicts related to compliance with health measures:

(…) But it's true that there are people who abused it, who couldn't go out, especially boys, young people who went out, who couldn't go out, and precisely, COVID grew thanks to these young people who knew they couldn't go out, and then they went out. [SAF017—Parent]

But it's still difficult to ask young people of our age to stop doing all that. To be able to go out and all. While it's a bit our…It's the age when you go out, you enjoy yourself and all. And we still enjoyed it despite the health orders and all that. [SAF019—Child]

Parents emphasized the difficulties associated with managing the anxiety of those around them and their fears for the health of their loved ones.

The study also highlighted diverse experiences regarding transitions in participants' lives linked to their children's life stage, changes in employment, and the transition from pediatric to adult care. Some parents conveyed restrictive beliefs regarding their child's capability of autonomy in managing their illness, like taking medication in time without supervision, and some children mentioned difficulties related to this aspect, as the fear of managing their illness alone. These difficulties seemed to have been exacerbated by the pandemic. Additionally, young people expressed concerns about their future, especially those with chronic illnesses, wondering whether they could start a family or pursue a profession, given their health condition. Young people with acute illnesses reported a strong sense of injustice bonded for example with the limitations of seeing friends to protect themselves or their sibling.

In response to the challenges posed by the pandemic, some participants adopted negative coping strategies, including increased substance consumption and excessive screen use. Hyperactivity, motivated by the fear of facing the difficult reality of families with children with chronic illnesses, was also mentioned. Some participants were aware of resources such as support groups but lacked the time to use them or chose not to use them as sometimes there were doubts about the utility of this kind of resources in regard of their needs. Despite these challenges, some families reminisced about the lockdown with a sense of nostalgia, emphasizing that it had strengthened family bonds.

Several personality traits emerged during the interviews, indicating the internal resources mobilized by the participants, such as resilience, optimism, and adaptability. Participants tended to contextualize their own situation and adopt positive coping strategies, such as doing activities in family or with friends online. Positive experiences related to a child's illness, such as transplant, stabilization or remission, had a positive impact on their experience, acting as a catalyst for the situation. Notably, in our sample, all the children showed stabilization or improvement in their health. Participants' previous experiences seem to have been positive elements that promoted the mobilization of adaptation strategies.

### Family level

Participants discussed the history of their children's illnesses, including treatments, medical care, and repercussions for the family. The illness significantly impacted family life, irrespective of the pandemic, and the question of death loomed in the interviews. The illness took center stage, affecting all family members, including siblings. Parents and siblings were willing to make sacrifices for the sick child, for example when a child has had cancer and the parents lived only for this child, sometimes neglect their social lives or even expressing a desire to take on the illness themselves. All aspects of family life were affected, including social, educational, professional, recreational, holiday, and daily routines. Hospitalizations, especially prolonged ones, also disrupted family functioning.

### Family isolation

The enforced lockdown during the pandemic had a major impact, bringing some families closer while isolating them from outside. Families' experiences during this period appeared to be strongly ambivalent in the interviews. As many supra-systems (businesses, schools, health care institutions, leisure activities, etc.) closed, the home had to absorb them, forcing parents to assume multiple roles, which sometimes proved to be a real challenge. This situation could lead to a loss of structure or, conversely, an excess of structure. Parents potentially found themselves performing technical procedures in place of health care professionals.

My father, he was scared. He was afraid that when he injects me intramuscularly, well, that he…he…it's not easy to inject his daughter either. But he had no choice. But he trembled. At first, right at the beginning. After that, it improved. [SAF004—Child]

While some appreciated an increased sense of control, it was a source of concern for others. Some families regretted the loss of external assistance, such as housekeeping aids, adding these aspects to their workload. This additional workload was compounded by the daily challenges of dealing with the illness. This aspect can also be found in the fact that for some interviewed families, COVID-19 was just one element among others in their experiences and ultimately held a secondary place. Some young people may have associated the closure of supra-systems with the disappearance of a sanctuary, such as a school, within families where an oppressive atmosphere prevailed.

### Hospitalization during COVID-19

Hospital visitation restrictions during COVID-19 pandemic caused suffering, especially for parents and siblings, who sometimes felt excluded from care. Hospitalizations resulted in the absence of the child from the home and, in some cases, the absence of one or both parents. Siblings appeared to be collateral victims of the situation, as their own needs were often set aside to focus on the crises of hospitalization and illness in general. Siblings also expressed fear of bothering nursing staff with their own problems.

### Family structure

Participants often mentioned elements related to the roles they assume within their families or those assigned to them by society, based on their gender, age, and profession. Parents see themselves as caregivers and are willing to do anything to take care of their sick child. They reported a fear of being ill and being unable to care for children, especially sick children, as well as concerns about the risk of a shortage of materials or medications to care for their child.

And yes, I'm afraid that if I catch COVID, I would die with it. Because I'm kind of the family's driving force. Yes, it's super stressful. But I'm lucky. [SAF010—Parent]

Several parents mentioned that they acted as safeguards during the introduction of the health measures. Notably, siblings also highlighted the protective role they assumed in shielding their brother or sister from COVID-19.

Parental couples experienced an imbalance in their dynamics due to hospitalizations and lockdown. The burden of illness also influenced the dynamics of the subsystems, particularly that of the couple. This phenomenon seemed to follow two scenarios presented repeatedly by families: either one parent bore the entire burden (mostly the mother in this sample), or they established a relay system. In the case of blended families, this dynamic became particularly complex during the lockdown, with questions about the role of stepparents.

Social determinants played a significant role, with precariousness negatively impacting the pandemic experience, while favorable socioeconomic status offered some protection. Having access to sufficient indoor or outdoor space, a structured routine, and technology (phones, computers, consoles, etc.) were beneficial factors.

Participants identified their roles as resources, including the increasing autonomy of children in managing their illness, thus relieving their parents and gaining independence, the feeling of being a health care professional for some parents, and the support of siblings, who served as a reassuring presence “in the world of adults” during hospitalizations.

Yeah, well, I couldn't go see him. And so, it was sad because I like to see my brother, anyway. But also, just for him, you know. It means that there were…Because I know that when I go to visit him, he likes it a lot because then there's someone a bit his age, you know. It's not just him and adults. And so, I knew that it was two weeks where there was…Well, no one, you know. [SAF009—Sibling]

Extended family and pets also served as significant resources. Family determinants seemed to influence how participants experienced the pandemic and illness. Families with multiple members with health problems, for example, parents with a chronic disease, had a unique perspective of the illness. For example, their own illness seemed not so important in comparison with the health of their child. Additionally, the type of illness that the child had strongly influenced the pandemic experience. Families with children with chronic illnesses had greater experience and an established routine, while families whose children were diagnosed with an acute illness during the pandemic faced two new challenges. Participants also showed disparities in their level of health literacy (vocabulary linked with the specific illness, understanding of the results, knowledge of the hospital structures and operations), which can be a resource if the level is high but also a challenge if it is low.

Families shared their intrafamily values, emphasizing hope in most interviews, which was often tied to spirituality. However, it was sometimes shaken, especially at the time of the diagnosis.

Because I said if God did this to my daughter, well, I don't understand anything. So, we said we believe in God, we believe in him, and I still believe. But I don't understand why things like this can happen to children. And the question I've always asked myself is why didn't it happen to me and it happened to my daughter. [SAF017—Parent]

This citation also showed that the decentering of oneself, as reported by many parents and siblings, is a common value within these families, as is communication.

Families expressed needs consistent with health care interventions but also encountered difficulties during the transition from pediatric to adult care. Differences in opinions regarding the vaccine affected trust in the health care system. Interprofessional teams were perceived as a significant resource for care support and disease self-management. Some participants turned to integrative medicine approaches to alleviate disease symptoms.

### Community level

A significant number of parents raised concerns about insufficient clarity in health guidelines, highlighting the persistent confusion regarding practices to adopt when dealing with specific health issues. This uncertainty exacerbated the challenges faced by families, particularly due to frequent routes to various health care institutions and variable hospitalization durations based on specific health problems. As some treatments were limited to certain regions, participants were forced to travel several hours to visit their sick loved ones. While some parents had to deal with unsympathetic employers regarding their child's illness, pressuring them about parental leave, others praised the flexibility of their employers and considered them as valuable support.

If it were to happen again, that’s it. I wouldn't do it. I wouldn't do it. I would take those darned 14 weeks^1^, to which we are entitled as parents. That's it. And he would stop…I felt guilty. [SAF013—Parent]

The hospital, considered a strictly enclosed place, was where traumatizing diagnoses were often made. A poignant example is a family whose child received a life-threatening diagnosis during the pandemic. The announcement took place when one parent was quarantined due to COVID-19, intensifying their distress. The other parent had to face this terrible news alone with their child, and the pain of that period remains palpable, even though the event dates back more than a year and the child is now in remission.

Experiences related to hospitalizations varied. Some participants highlighted positive aspects, such as low attendance in pediatrics during the pandemic and high-quality communication with some health care professionals. However, others denounced issues with access to necessary amenities and gaps in parental care. The military presence at the hospital entrances also left a striking impression.

About home care, it sometimes elicited feelings of intrusion, particularly the impression of allowing COVID-19 in one's home. In specific cases, families mentioned that nurses were occasionally poorly trained in very specific technical care, adding an extra layer of anxiety for parents, children, and adolescents. Communication gaps also led to misunderstandings within families, sometimes affecting trust in health care teams. For instance, insufficient communication about the procedure of home care or about impacts of COVID-19 on specific illness led to stress in the families. However, home care nurses were generally perceived as valuable resources, offering close support and establishing significant trust with families. Their services helped to minimize hospitalizations during the crisis, thereby reducing school absenteeism.

Interprofessional collaboration was highlighted as essential, with communication perceived as crucial to ensuring care continuity and the integration of different therapies. Several participants mentioned good communication in hospitals, despite restrictions during the pandemic, with clear information and support by answering questions. Most participants also described a form of advocacy from health care personnel, going beyond measures for the well-being of parents and children in the hospital. However, these statements should be nuanced, as they seemed to depend on individuals and institutions.

Some participants highlighted that simply following health guidelines facilitated their daily functioning by providing an understandable and shared isolation routine for everyone. The collective experience of the pandemic was also considered an opportunity to raise public awareness of the realities experienced by these families for many years.

Yes, everyone was on the same boat. We all took precautions because we didn't want to feel responsible. [SAF027—Parent]

Some families benefited from external support, such as home deliveries of medicines or food, particularly because they appreciated avoiding crowded places during the pandemic. Social support was crucial for precarious families. Participants also addressed challenges related to protective equipment, noting adaptation problems, especially for children and people with certain illnesses; adjustments needed for new habits; and sparking discussions about the protective or irritating nature of masks.

### Family needs

Parents emphasized the necessity for support, in terms of human and material resources, in transitioning their children toward empowerment, as well as guidance for managing care. They expressed the need for emotional support and attentive listening from health care professionals, highlighting the importance of clarifying information to counteract media-induced anxiety. Some mothers also mentioned more concrete needs, such as assistance with household chores and priority access to home deliveries. They underscored the need for a better hospital organization, particularly concerning the care of parents and siblings. Finally, they expressed the need to recognize their role as caregivers, highlighting the difficulty in recharging and taking vacations.

And then, we would like to be able to rely on…Really rest. Without worries. That would be your wish, there.—If you had a wish.—Yes. It's…It's to have real vacations. [SAF013—Parent]

## Discussion

The results of this study shed light on the experiences of children and adolescents who received pediatric home care during the COVID-19 pandemic as well as their family, including the siblings. The results were heterogenous and depended on various factors as the age, role or socio-economical of the participants. So opposite results were obtained among others on subject as the benefits of the semi-confinement, of vaccines, of modification and multiplication of the roles linked with the pandemic and its impact on the society and the supra-systems. The experiences were taking place at different levels and were deeply embedded in the different systems that comprise their lives. The experiences, resources and challenges were not always directly linked to the pandemic but reflected a more generalized experience of these families.

The findings of this study are consistent with what we have observed in the literature. Indeed, families reported a shift and an increase in the burden associated with the care of children and adolescents (13). This could be linked to the disruption of continuity of care in certain situations (14). The ambivalent relationships towards hospitals (16) and home care teams (19, 20) could also underpin this. The psychosocial and economic impacts related to the isolation of families (18) emerged in the interviews. We therefore observed significant consequences of the pandemic on the functioning and well-being of families, as well as on the management of children's and adolescents' illnesses.

Our findings highlight that the COVID-19 pandemic has notably highlighted the generation shock, with noticeable differences in how various age groups experienced this crisis. Young people often felt the weight of restrictions and social distancing, and the impact on their education, while adults seemed to have been more affected by the situation of their family in general than by the pandemic itself. Families we interviewed faced challenges at the individual, familial, and community levels. They also shared their resources, which were equally comprehensive and originated from the same levels. The COVID-19 pandemic has exacerbated these families' vulnerability but also demonstrated their resources. The COVID-19 and its incidences act as a close up on pre-existing difficulties in the daily life of families with chronic or acute illness; the challenges associated with managing chronic or acute illnesses in children are numerous and complex. They can vary depending on the type of illness and prior health experience of the family. These families must address constant health constraints, concerns, and questions related to their child's health, as well as make significant adjustments to their lifestyle. However, the personality traits of the participants evidently tinted their lived health experiences. For example, for children with chronic illnesses and their families, the age at which the illness is diagnosed can impact acceptance. Children who develop chronic illnesses at a young age may develop resilience and ease of acceptance because they grow up incorporating the illness into their identity. However, this resilience does not minimize the challenges they face.

This element highlights the profound impact that children's chronic illnesses have on families and raises important questions about how families manage these difficult situations and adapt to the many challenges they face. Thus, questioning the role of society in supporting these families and the resources available to help them daily is important. In the pandemic context, health restrictions have made access to care more difficult, school closures have impacted the education of sick children, notably those with anxiety who worried about going back to school which resulted on a school dropout and fear of infection has been particularly worrisome. Society must consider the specific needs of these families in crisis situations and offer them adequate support like priorities food delivery or providing psychological support for families, including siblings.

According to our findings, gender roles within families also play a crucial role. Mothers are often perceived as having the primary responsibility for caring for sick children, which can impact their economic status, as they may sometimes be forced to reduce their professional activity to take care of the child, as well as impacts on their mental and physical health. According to Shattnawi et al. (2023), children with chronic illnesses are sometimes limited in their ability to perform certain daily actions and need the help of a caregiver (42). Therefore, parents must spend a certain number of hours assisting their child, which can result in a decrease in the time allocated to paid work. This burden particularly falls on women (43). Moreover, women caregivers feel the associated care burden more significantly (44). The same would be true for caregivers with poor health. Care coordination is essential in the context of chronic illnesses, and health care professionals must collaborate closely with families to ensure effective and holistic care. Indeed, a study conducted in Iceland reported that a systemic nursing intervention can improve participants' quality of life, perceived family support, or perceptions of the quality of the health care system (45). The participants in our study repeatedly stated that they considered themselves caregivers of their children. Additionally, extended family members appear to become caregivers for these caregivers. As caregivers, family members play an essential role in the care of the child and need emotional and practical support. These needs seem to differ depending on the level of education of caregivers. Indeed, in a scoping review, the authors highlighted that parents with a secondary education level desire more practical support, while parents with a university education level are more likely to look for psychological support (46). Nevertheless, these roles must be recognized, supported, and accompanied by official bodies. Significant progress has already been achieved in our country; however, it remains insufficient to provide relief to families.

Social determinants of health, such as socioeconomic status (SES), significantly impact the health experience of families. According to a Chinese study, SES has a particularly pronounced impact on physical health but less so on mental health (47). This study highlights the impact of lifestyle on both physical and mental health. This element appears to be a mediator between SES and health status. Another mediator between SES and health status is health literacy. According to Stormacq et al. (2019), health literacy plays a major role in health disparities among people with different SESs (48). However, the participants in these studies have shown significant differences in health literacy, which can impact the quality of their caregiving for themselves or their loved ones. Therefore, supporting vulnerable families to ensure that they can access the necessary care and resources is crucial, by providing written and clear information of procedures and existing resources for instance.

Ultimately, family solidarity and resilience play major roles in managing children's chronic and acute illnesses. Families support each other and create support networks. These support networks are crucial to families and contain several elements. Indeed, Chakraborti et al. (2021) emphasize in their scoping review that support networks (even those artificially created for research) allow for “creating a shared experience,” “promoting optimism and empowerment,” “learning,” “developing social opportunities,” “increasing the sense of belonging,” and “developing emotional support and psychological well-being.” Therefore, these networks enable families to provide support at various levels. Similarly, the authors argued that these groups serve as extensions of individual strategies. An important goal for society is to recognize and support the crucial and consistent work of these families. A systemic approach to care, which recognizes the central role of the family, is essential for improving the quality of life of sick children and their loved ones and taking care of the family as a unit. A better recognition of their role as caregivers on a political and economic level would help, as well as the visibility of their crucial work.

### Strengths and limitations

The strength of our study is that it successfully brought together the voices of sick children and adolescents, their siblings, and their parents during the COVID-19 pandemic. However, our study had some limitations. The participants often spoke in vague terms and admitted to being lost in their memories, creating chronological confusion when they recalled the different phases of the pandemic, government measures, the arrival of the vaccine, and school and institutional organization. A relevant observation is that younger children had more difficulty providing clear memories than their elders and parents, sometimes making the answers less detailed. A large majority of the interviews likely had a “therapeutic” effect on the participants because of the tone and words used, which evolved during the interviews. Indeed, several people thought that they had nothing to report about this period and were surprised by the evolution of their discourse. On many occasions, very strong emotions emerged from these discourses. The participants expressed satisfaction with being able to express what they had experienced at that time. Future projects should be conducted to collect more sibling testimonials to complement the results of this study, as well as on families whose child health is not stabilized to access real time emotions and experience and not emotion blurred by time.

## Conclusions

To our knowledge, this study is the first to give voice to families in the Vaud region to understand their health experiences during the COVID-19 pandemic. The participants freely shared the different challenges they faced, as well as the numerous resources they had at different levels. Most of the interviewed families exhibited an extraordinary degree of resilience and an ability to manage extraordinary crisis situations. However, this resilience did not decrease the suffering they experienced during this period, and lessons must be learned to better prepare for future pandemics. Meanwhile, some measures must also be quickly implemented to counter the long-term deleterious effects of the pandemic, especially regarding the health of children and adolescents particularly in terms of psychosocial support for families. A better focus should be made on siblings to take care of them as they are to often the great forgotten ones.

## Data Availability

The raw data supporting the conclusions of this article will be made available by the authors, without undue reservation.

## Footnotes

^1^According to the Federal Social Insurance Office of Switzerland: "From July 1, 2021, parents who have to interrupt or reduce their gainful activity to care for a minor child seriously affected by illness or accident will be able to take 14 weeks' paid leave" (41).
