# Supplementary material for: The health experience of children, adolescents and their families during the COVID-19 pandemic: an exploratory qualitative study in pediatric homecare
Source: Front Pediatr. 2025 Jun 20;13:1492433. doi: 10.3389/fped.2025.1492433 (PMC12226480; doi:10.3389/fped.2025.1492433)
Supplement: Supplementary file 2 [file Supplementaryfile2.docx]

| Contextual | |
| --- | --- |
| Pandemic experience/memories | Refers to historical elements and memories related to the pandemic itself. |
| Fears for the future | Refers to elements linked to concerns about the future. |
| Family system | |
| Roles | Refers to the different roles in the family (child and family). |
| Family functioning | How the family works in everyday life |
| Family description | Refers to family picture |
| Personality traits | Of the individual. Not if it concerns someone other than oneself. |
| Needs | Refers to the various needs not clearly expressed by families (child and family) (future elements) |
| Requests | Refers to the nature of requests or types of requests (reasons for consultation). Concrete elements |
| Challenges | Challenges faced by families (child and family). Can refer to emotions (e.g. communication). Past elements. |
| Facilitators/Resources | Elements that facilitate the health experience and resources (child and family) |
| Constraining beliefs | Beliefs that impact, stop or hinder the family's health (child and family). |
| Facilitating beliefs | Beliefs that promote family health (child and family). |
| Values | Attributes or established values that impact family health (child and family) |
| Nursing team system | |
| Roles | Refers to the perception of each person's role in the work organization |
| Challenges | Refers to the perception of the challenges faced by the nursing team (including conflicts of values/loyalty). Past elements |
| Care environment | |
| Institution | Guidelines, operation, policy or organization |
| Equipment | Refers to the material elements required to carry out a treatment. |
| Spatio-temporal organization | Refers to the temporal elements, transitions and organization of the care environment. |
| Collaboration | |
| Interprofessional | Includes elements of collaboration between the multidisciplinary team, the nursing team and the family. |
| Vision | Refers to the shared or differing values and visions of multidisciplinary and interprofessional teams |
| Nurse intervention | |
| Target population | Refers to the people for whom the nursing intervention is intended. |
| Frequency | Refers to the frequency of interventions (how many times per day or per week). |
| Duration | Refers to the duration of interventions (in minutes or estimated time) |
| Stakeholders | Refers to people active in the intervention |
| Components | Refers to the nature of the nursing intervention (e.g. psycho-social, support, education, prevention, etc.). |
| Modalities | Refers to nursing intervention modalities such as videoconference consultation |

Appendix 1: Coding Matrix and definitions
